# Natural Polysaccharide-Based Nanoparticles Enhance Intracellular Delivery and Cytotoxicity of *Antrodia camphorata* in Breast Cancer Cells

**DOI:** 10.3390/ijms26178420

**Published:** 2025-08-29

**Authors:** Yu-Chen Tsai, Hiroki Miyajima, Ming-Yang Chou, Satoshi Fujita

**Affiliations:** 1 Department of Frontier Fiber Technology and Sciences, University of Fukui, Fukui 910-8507, Japan; 2 ROHER Technology Co., Taichung 41141, Taiwan

**Keywords:** *Antrodia camphorata*, nanoparticle, cytotoxicity, anticancer

## Abstract

*Antrodia camphorata* (AC), a medicinal fungus native to Taiwan, contains bioactive compounds such as triterpenoids with anticancer properties. However, their high lipophilicity results in poor aqueous solubility and limited bioavailability, restricting their therapeutic application. To address this issue, a nanoparticle-based delivery system was developed using chitosan, alginate, and hyaluronic acid to encapsulate AC extracts. AC-loaded nanoparticles (AC-NPs) with a particle size less than 100 nm improved drug solubility and facilitated intracellular accumulation. Assessment of cytotoxicity revealed that AC-NPs significantly and more effectively suppressed the growth of breast cancer cells than free AC extracts. After 72 h, IC_50_ values for MDA-MB-231 (triple-negative) and MCF-7 (estrogen receptor-positive) were 46.9 and 75.6 μg/mL, respectively, with greater sensitivity observed in MDA-MB-231 cells. AC-NPs exhibited minimal toxicity toward normal mammary epithelial cells (NMuMG), indicating good biocompatibility. Fluorescently labeled AC-NPs showed rapid, time-dependent uptake in both cancer cell lines. Particularly, MDA-MB-231 cells exhibited rapid internalization, whereas MCF-7 cells likely benefited from hyaluronic acid-mediated targeting of CD44 receptors. In conclusion, AC-NPs enhanced the solubility, cellular uptake, and anticancer efficacy of AC while maintaining biocompatibility, thereby suggesting their robust potential as nanocarrier platforms for breast cancer therapy.

## 1. Introduction

Cancer is a major public health concern worldwide. By 2025, it is estimated that breast, lung, and colorectal cancers will account for 51% of all new cancer cases in females, with breast cancer accounting for 32% [1]. Breast cancer cells are highly aggressive and can spread through the lymphatic system or blood circulation to the lymph nodes and distant organs, leading to metastatic breast cancer, which can be challenging or difficult and is life-threatening. Therefore, the development of effective and selective breast cancer drugs capable of inhibiting tumor growth and preventing metastasis is crucial for reducing mortality. The current standard treatments for breast cancer include surgery, radiotherapy, chemotherapy, hormonal therapy, and targeted therapy [2]. However, owing to the heterogeneity of breast cancer, treatment responses vary among subtypes. For example, MDA-MB-231 cells are triple-negative breast cancer (TNBC) cells, which are characterized by high invasiveness and metastatic potential. Given the lack of estrogen receptor (ER), progesterone receptor, and human epidermal growth factor receptor 2 (HER2), there are no effective targeted therapies for patients with TNBC, resulting in limited options and poor prognosis [3]. Furthermore, several conventional anticancer drugs have serious side effects such as bone marrow suppression, nausea, and hepatotoxicity. In contrast, MCF-7 cells belong to the ER-positive (ER^+^) breast cancer subtype, which is known to respond to hormonal therapy [4,5]. These cells exhibit low invasiveness and are frequently employed in drug screening for hormone receptor-positive breast cancer. However, long-term hormonal therapy can lead to drug resistance [6].

Given the limitations of current therapies for both TNBC and ER^+^ breast cancer, there is growing interest in identifying and developing novel therapeutic agents. One promising candidate is *Antrodia camphorata* (AC), a medicinal fungus native to Taiwan. Traditionally used to alleviate alcohol-related discomfort, fruiting bodies of AC have also been used to treat liver disease, poisoning, and hypertension [7,8]. AC contains various active compounds, including polysaccharides, triterpenoids, and maleic and succinic acid derivatives, which possess anti-inflammatory, anti-mutagenic, and anticancer properties, highlighting its potential as a candidate for biomedical applications. AC has been shown to inhibit the growth of several cancer cell types, including breast [9], bladder [10], cervical [11], and liver cancers [12]. Among its bioactive compounds, triterpenoids play a central role in mediating anticancer, anti-inflammatory, hepatoprotective, and antioxidative effects [13]. Recent original studies further highlight the anticancer potential of naturally derived triterpenoids. A triterpenoid-rich fraction from *Forsythia suspensa* leaves—mainly ursolic, oleanolic, and betulinic acids—induces mitochondrial apoptosis and cytotoxicity in MCF-7 and MDA-MB-231 cells [14]. One of the triterpenoids isolated from *Dysoxylum malabaricum* shows potent cytotoxicity against MDA-MB-231 cells with apoptotic features, supporting the rationale for focusing on natural triterpenoids in this study [15]. However, the high lipophilicity of these compounds leads to poor aqueous solubility, which severely limits their bioavailability and stability. Despite numerous efforts, improving the solubility of triterpenoids remains a major challenge owing to their rigid and hydrophobic chemical structures [16,17].

Various nanocarrier systems have been explored to address this issue. Chitosan is a natural cationic polymer that can be derived from crustacean shells, cellulose, or fungal fermentation, and has attracted considerable attention as a potential drug carrier. Chitosan comprises *N*-acetyl-d-glucosamine and d-glucosamine units [18], is biodegradable and biocompatible, and lacks toxic side effects [19]. Chitosan becomes positively charged in acidic environments, thereby enhancing its interactions with negatively charged cell membrane receptors and promoting intracellular uptake of nanoparticles (NPs). Moreover, chitosan can be used to encapsulate hydrophobic drugs, thereby improving their solubility and stability. The drug release rate can be adjusted by regulating the degree of cross-linking with sodium tripolyphosphate, thereby enabling sustained release [20]. In combination with hyaluronic acid (HA), chitosan can achieve targeted delivery mediated via specific receptors (such as CD44).

We applied nanocarrier technology to enhance the solubility, cellular uptake, and biological activity of AC [21,22], to improve its therapeutic potential in breast cancer treatment. Various strategies have been proposed to improve the aqueous solubility and bioavailability of triterpenoids, including NP formulations, solubilizers, and chemical modifications [13]. Among these, NPs are particularly promising owing to their ability to enhance drug solubility, prolong circulation, and enable controlled and sustained release [23,24,25]. In addition, NPs can enhance therapeutic efficacy by selectively accumulating at tumor sites through active or passive targeting, thereby reducing off-target effects [26,27]. Nanocarriers such as lipid-based particles, polymeric systems, and inorganic nanomaterials have been extensively studied for these purposes and demonstrate considerable potential in triterpenoid-based cancer therapies [28,29]. To evaluate the anticancer effects of encapsulated AC, we used two breast cancer cell lines, MDA-MB-231 and MCF-7, which represent the TNBC and ER^+^ subtypes, respectively. This dual-cell approach provides a comprehensive understanding of the effects of encapsulated AC in different breast cancer types, with a focus on cell viability, apoptosis, and pinocytosis. Additionally, we prepared fluorescently labeled nano-AC to examine its intracellular localization and analyzed its cellular uptake using confocal microscopy and flow cytometry. These findings can contribute to a better understanding of the anticancer mechanisms of AC and support its potential applications in breast cancer therapy.

## 2. Results

### 2.1. Characterization of AC-Loaded NPs (AC-NPs)

#### 2.1.1. Surface Morphology and Particle Size of Synthesized AC-NPs

**Figure 1:**
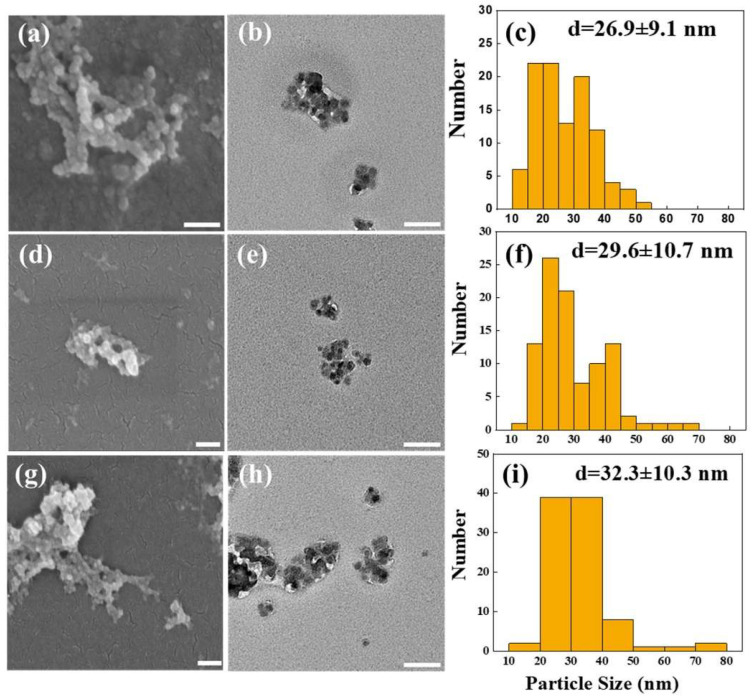
Morphological and size characterization of nanoparticles. (**a**–**c**) Unloaded-NPs, (**d**–**f**) AC8-NPs, and (**g**–**i**) AC16-NPs. (**a**,**d**,**g**) SEM images, (**b**,**e**,**h**) TEM images, and (**c**,**f**,**i**) corresponding particle size distributions derived from TEM analysis. All scale bars represent 100 nm. AC, *Antrodia camphorate*; NPs, nanoparticles; SEM, scanning electron microscopy; TEM, transmission electron microscopy.

According to the scanning electron microscopy (SEM; Figure 1a,d,g) and transmission electron microscopy (TEM; Figure 1b,e,h) images, unloaded-NPs (UL-NPs) and AC-loaded NPs (AC8-NPs and AC16-NPs) displayed a spherical morphology, moderate size uniformity, and aggregation across samples. The particle size distribution based on TEM images is shown in Figure 1c,f,i. UL-NPs had an average diameter of 26.9 ± 9.1 nm (range: 15–55 nm). AC loading slightly increased size, with AC8-NPs reaching 29.6 ± 10.7 nm and AC16-NPs reaching a size of 32.3 ± 10.7 nm (range: 15–80 nm).

SEM/TEM imaging and particle-size analysis confirmed uniform, stable nanoparticles. To evaluate long-term stability, SEM was repeated at 1, 6, and 10 months post-preparation. As shown in Figure S1, both AC8-NPs (a–c) and AC16-NPs (d–f) maintained their morphology with no appreciable shape changes. Mean diameters were 29.6, 26.7, and 27.8 nm for AC8-NPs, and 32.3, 33.5, and 30.2 nm for AC16-NPs, indicating minimal variation. These results support that AC-NPs remain stable for at least 10 months at room temperature.

#### 2.1.2. Fourier-Transform Infrared (FTIR) Analysis

**Figure 2:**
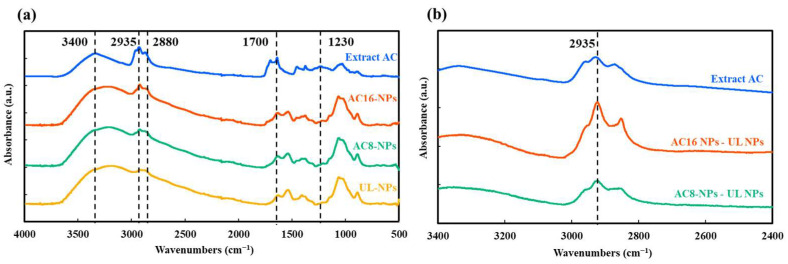
(**a**) FTIR spectra of AC extract, unloaded (UL)-NPs, AC8- NPs, and AC16-NPs. (**b**) FTIR spectra of AC8-NPs and AC16 NPs after background subtraction of UL-NP, highlighting characteristic peaks attributed to the encapsulated AC. AC, *Antrodia camphorate*; NPs, nanoparticles; FTIR, Fourier-transform infrared.

FTIR analysis revealed that the spectrum of AC-NPs closely resembled that of UL-NPs but differed from that of the AC extract (Figure 2a). In the FTIR spectrum of the UL-NPs, the absorption peaks at 1374 and 1540 cm^−1^ could be attributed to the N–H bending vibration of the Amide II bond and the deformation vibration of the –NH_2_ functional group from chitosan, respectively [30,31,32]. In addition, a prominent peak at 1029 cm^−1^ corresponded to the C–O–C stretching vibration of the polysaccharide backbone of HA, serving as one of its characteristic structural peaks [30,33]. For alginate, two distinct peaks could be observed at 1633 and 1405 cm^−1^, which were attributed to the asymmetric and symmetric stretching vibrations of carboxylate groups (COO^−^), representing characteristic absorptions of the carboxylate groups in alginate [31,32]. The AC extract exhibited characteristic absorption peaks consistent with previous reports [34,35,36]: a broad OH stretching near 3400 cm^−1^, aliphatic C–H stretching at 2935 and 2880 cm^−1^, and a strong C=O stretching around 1700 cm^−1^. The region from 1230 to 1000 cm^−1^ exhibited multiple peaks associated with C–O–C and C–O in polysaccharides. Notably, the ester/carboxylic acid C=O peak at 1704 cm^−1^ in the AC extract shifted slightly, changing in intensity in both AC8-NPs and AC16-NPs, thereby suggesting potential interactions between AC and the carrier material. Moreover, the intensity of the aliphatic –CH_2_– asymmetric stretching peak at 2935 cm^−1^ increased in the AC-NPs compared with that in UL-NPs. This change was determined by subtracting the UL-NPs spectrum from the background data (Figure 2b).

#### 2.1.3. Determination of Drug Loading (DL%) and Encapsulation Efficiency (EE%)

**Figure 3:**
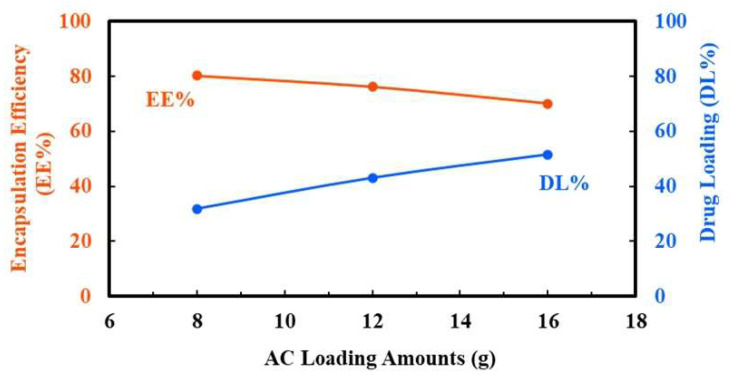
Encapsulation efficiency (EE%) and drug loading (DL%) of nanoparticles prepared as a function of AC input amounts (8, 12, and 16 g, respectively). The graph illustrates the inverse relationship between EE% and DL% with increasing AC loading during nanoparticle preparation.

To determine the effect of incorporating varying drug amounts on the coating capacity of NPs, the DL% and EE% were measured upon adding 8 g, 12 g, and 16 g of the drug, respectively (Figure 3). The DL% increased with increasing drug input, yielding values of 31.9, 43.1, and 51.5%, respectively. In this study, 8 g of AC was initially added, and 6.6 g was successfully encapsulated in the NPs. In contrast, the EE% decreased progressively to 80.3, 76.2, and 70.0%, respectively. Notably, AC8-NPs exhibited the highest EE% while maintaining a reasonable DL%. Accordingly, AC8-NPs were selected as the optimal formulation for subsequent cellular experiments.

### 2.2. In Vitro Cytotoxicity Analysis

**Figure 4:**
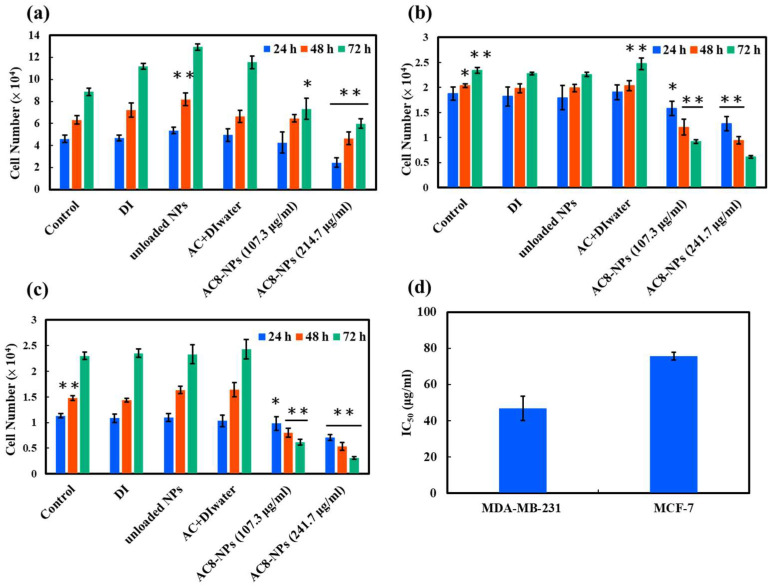
Cell viability analysis using WST assay for (**a**) NMuMG, (**b**) MDA-MB-231, and (**c**) MCF-7 cells treated with deionized (DI) water, unloaded (UL)-NPs, AC+DI water, and AC8-NPs at two concentrations (107.3 µg/mL and 241.7 µg/mL) for 24, 48, and 72 h. Control samples were untreated. Data represent the mean ± standard deviation (SD) (*n* = 6) from three independent experiments. * *p* < 0.05 and ** *p* < 0.001 compared to the DI water group. (**d**) IC_50_ values of AC8-NPs after 72 h incubation of MDA-MB-231 and MCF-7 cell lines. AC, *Antrodia camphorate*; NPs, nanoparticles.

To examine the anticancer potential of AC-NPs, normal mammary epithelial cells (NMuMG) and two breast cancer cell lines (MDA-MB-231 and MCF-7) were treated with the free AC extract, AC-NPs, or UL-NPs for 24, 48, and 72 h (Figure 4). The results revealed that the free AC extract in water exerted minimal inhibitory effects, which could be attributed to its extremely poor aqueous solubility owing to its hydrophobic nature. This suggests that poor solubility severely limits the bioavailability and therapeutic potential of free AC extracts when used alone.

In contrast, AC-NPs exhibited significantly improved AC dispersion and stability in the aqueous phase, which facilitated cellular uptake and resulted in enhanced drug release and the induction of cancer cell death. After 72-h treatment, the survival rates of MDA-MB-231 and MCF-7 cells decreased significantly in a dose- and time-dependent manner, indicating the potential of AC-NPs as effective anticancer drug carriers.

In addition, as shown in Figure 4a, even at a high concentration (107.3 μg/mL), AC-NPs only partially inhibited the proliferation of NMuMG cells, with cell numbers increasing progressively over time. This suggests that AC-NPs exhibit good biocompatibility and are well tolerated by normal cells. Interestingly, treatment with lower concentrations (13.4, 26.8, and 53.6 μg/mL), as shown in Figure S2a, promoted cell proliferation, indicating the potential positive regulatory effects of AC-NPs on normal cellular function.

Further analysis of Figure S2b,c revealed that both cancer cell lines exhibited significant growth inhibition and cell death when treated with AC-NPs at a concentration of 107.3 μg/mL. These results confirm that the anticancer effects of AC-NPs are dependent on both concentration and exposure time. Based on the 72-h data (Figure 4d), the calculated IC_50_ for MDA-MB-231 was 46.9 μg/mL, lower than that for MCF-7 (75.6 μg/mL), indicating that MDA-MB-231 cells were more sensitive to AC-NPs.

### 2.3. Cellular Internalization of AC-NPs

**Figure 5:**
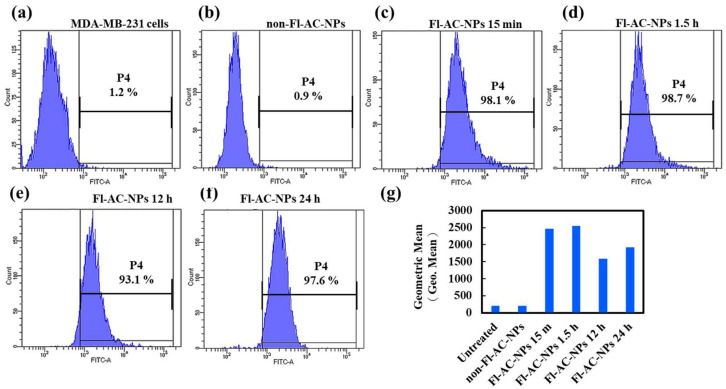
Flow cytometry analysis of cellular uptake of Fl-AC-NPs by MDA-MB-231 cells. Histograms represent fluorescence intensity in (**a**) untreated cells, (**b**) cells treated with NPs for 1 h, and cells treated with Fl-AC-NPs for (**c**) 15 min, (**d**) 1.5 h, (**e**) 12 h, and (**f**) 24 h. (**g**) Quantification of fluorescence area based on geometric mean, indicating time-dependent internalization of Fl-AC-NPs. AC, *Antrodia camphorate*; Fl-AC-NPs, fluorescein-labeled AC-NPs; NPs, nanoparticles.

Flow cytometry was used to evaluate the cellular internalization and time-dependent uptake of fluorescein-labeled AC-NPs (Fl-AC-NPs) in two breast cancer cell lines: MDA-MB-231 and MCF-7. In MDA-MB-231 cells, Fl-AC-NPs were rapidly incorporated into cells within a short period. A distinct fluorescence signal was detected as early as 15 min (Figure 5c), indicating that NPs were internalized or adsorbed onto the cell surface. The signal further intensified after 1.5 h (Figure 5d). However, a decrease in fluorescence intensity was observed at 12 h (Figure 5e), possibly due to the intracellular transport, degradation, or clearance of the NPs. At 24 h (Figure 5f), the signal increased slightly, indicating possible re-accumulation or cellular adaptation via internalization and efflux mechanisms. Negligible fluorescence was detected in the untreated control group (Figure 5a), confirming that the observed signals were due to the Fl-AC-NPs. These results appear to be quantitatively reflected in the geometric mean fluorescence intensity values (Figure 5g). The progressive increase in fluorescence intensity over time indicated efficient and time-dependent cellular uptake of the NPs.

**Figure 6:**
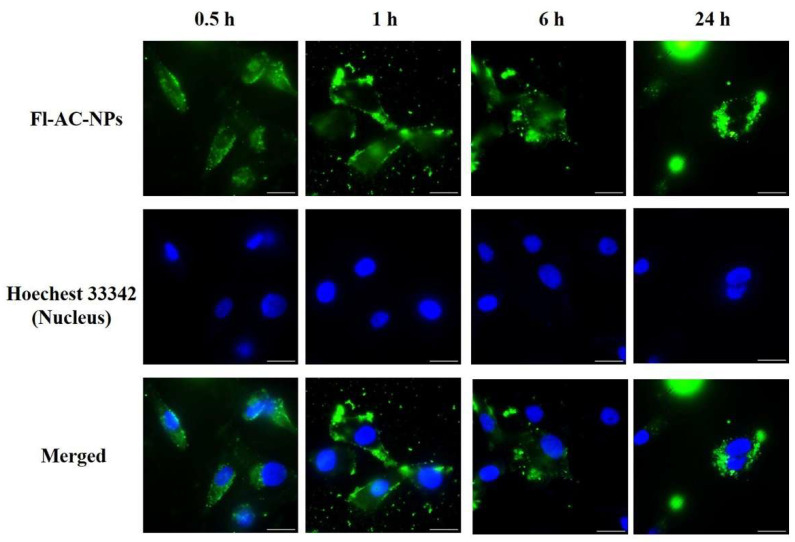
Fluorescence microscopy images of MDA-MB-231 cells incubated with Fl-AC-NPs at 37 °C for 0.5, 1, 6, and 24 h. Green fluorescence indicates captured Fl-AC-NPs, while blue fluorescence indicates nuclei stained with Hoechst 33342. Scale bars = 20 μm. AC, *Antrodia camphorate*; Fl-AC-NPs, fluorescein-labeled AC-NPs; NPs, nanoparticles.

Fluorescence microscopy further supported these findings (Figure 6), demonstrating notable cytoplasmic distribution of Fl-AC-NPs in MDA-MB-231 cells, confirming their effective internalization. These results indicated that Fl-AC-NPs were rapidly internalized by MDA-MB-231 cells and that their uptake exhibited time-dependent dynamics.

**Figure 7:**
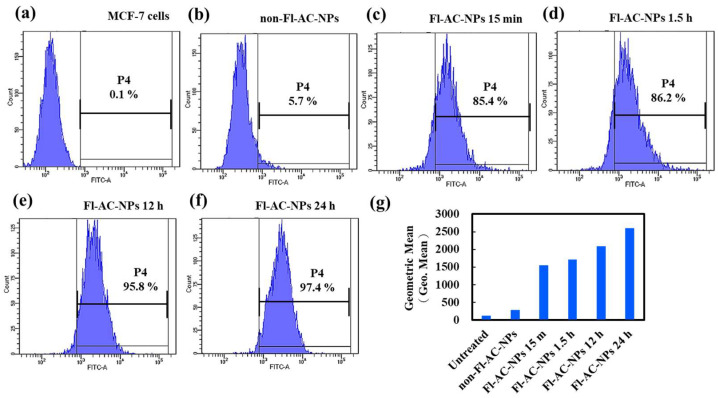
Flow cytometry analysis of cellular uptake of Fl-AC-NPs by MCF-7 cells. Histograms show fluorescence intensity in (**a**) untreated cells, (**b**) cells treated with NPs for 1 h, and cells treated with Fl-AC-NPs for (**c**) 15 min, (**d**) 1.5 h, (**e**) 12 h, and (**f**) 24 h. (**g**) Quantification of cellular uptake based on the geometric mean of fluorescence area, indicating time-dependent internalization of Fl-AC-NPs. AC, *Antrodia camphorate*; Fl-AC-NPs, fluorescein-labeled AC-NPs; NPs, nanoparticles.

Likewise, flow cytometric analysis of MCF-7 cells (Figure 7) demonstrated a time-dependent uptake pattern. The fluorescence intensity histograms (Figure 7c–f) revealed a progressive rightward shift with increasing incubation time, indicating a continuous increase in intracellular fluorescence intensity and, hence, NP internalization. This trend was quantitatively reflected in the geometric mean fluorescence intensity (Figure 7g), suggesting a cumulative uptake effect.

**Figure 8:**
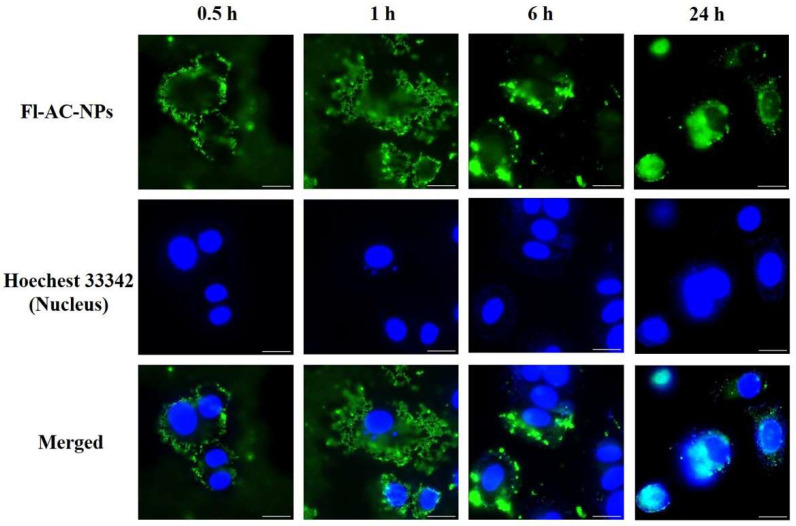
Fluorescence microscopy images of MCF-7 cells incubated with Fl-AC-NPs at 37 °C for 0.5, 1, 6, and 24 h. Green fluorescence indicates captured Fl-AC-NPs, and blue fluorescence shows nuclei stained with Hoechst 33342. Scale bars = 20 μm. AC, *Antrodia camphorate*; Fl-AC-NPs, fluorescein-labeled AC-NPs; NPs, nanoparticles.

Fluorescence microscopy confirmed the internalization pattern in MCF-7 cells (Figure 8). From 15 min to 6 h, fluorescence was predominantly localized around the cytoplasmic periphery in a granular form, suggesting cytoplasmic retention. At 24 h, the fluorescence overlapped with DAPI-stained nuclei, indicating co-localization and suggesting that some NPs may have penetrated the nucleus or accumulated at the nuclear membrane.

Overall, these results demonstrated that Fl-AC-NPs undergo rapid internalization in both MDA-MB-231 and MCF-7 cells in a time-dependent manner and may reach the nuclear region after extended exposure.

## 3. Discussion

### 3.1. Design of AC-Loaded NPs

In the current study, we examined the anticancer potential of AC-loaded NPs. Although the anticancer activity of AC has been reported [9,10,11,12], its clinical application is limited owing to its poor aqueous solubility and low bioavailability [16,17]. To corroborate this, we assessed solubility by adding AC extract to DMSO and deionized water; it dissolved readily in DMSO but poorly in water (<200 μg/mL). Herein, we applied nanotechnology to improve the solubility and cellular uptake efficiency of AC, thereby validating its inhibitory effect on breast cancer cells and providing a potential strategy for its application in cancer therapy.

We demonstrated that AC was successfully encapsulated in chitosan, substantially enhancing its poor aqueous solubility and bioavailability owing to its hydrophobicity. This enhancement could be attributed to the synergistic effects of noncovalent intermolecular interactions. Chitosan, a linear polysaccharide derived from chitin deacetylation, contains abundant amine (-NH_2_) and hydroxyl (-OH) groups that can form hydrogen bonds with the hydroxyl or carboxyl groups of AC components, creating a stable interaction interface [37,38]. Hydrophobic AC components, such as triterpenoids and fatty acids, may be embedded in the nonpolar regions of chitosan through hydrophobic interactions, promoting the formation of a stable nanocarrier structure [39,40].

### 3.2. Characterization of AC-Loaded NPs

According to the particle size analysis (Figure 1c,f,i), the average particle size of AC-NPs varied slightly with changes in the drug ratio but remained within a stable and moderate range. This suggests that the preparation method facilitated good reproducibility and control over particle size. Stable and moderate particle sizes help to inhibit aggregation and facilitate phagocytosis and tumor penetration, thus enhancing their potential as drug delivery systems [41]. When other physicochemical properties of the control drug-NP complexes are consistent, differences in particle size (e.g., 20, 50, and 200 nm) can markedly impact their biodistribution. Smaller NPs (20 and 50 nm) have shown greater efficacy in the passive targeting of tumor tissues. In particular, 20-nm particles exhibit faster tumor penetration and signal clearance, highlighting their superior performance in drug delivery [42].

In addition, FTIR analysis revealed that the NPs exhibited absorption peaks corresponding to the AC component (Figure 2a). These peaks, such as those attributed to the C–H stretching of triterpenoids or fatty acids, were consistent with known AC spectra. Although this suggests successful encapsulation, the presence of these signals alone does not conclusively confirm molecular embedding; rather, they support the possibility of physical inclusion or surface association of AC within the NPs, which also implies that the AC chemical structure is preserved during the encapsulation process and, hence, may help retain its pharmacological activity.

### 3.3. Formulation Optimization

As shown in Figure 2b, the drug-loading content increased with higher drug input, demonstrating favorable DL% capacity and tunability. To further evaluate this observation, we examined the effect of AC input levels (31.9–51.5%) and found that the EE% decreased significantly (from 80.3 to 70.0%). These results suggest that the NP system has a saturation threshold for drug encapsulation. When this threshold is exceeded, the surplus drug is less likely to be incorporated within the NP core and instead remains unencapsulated or adsorbed onto the surface. Similar observations have been reported by Hamdallah et al. (2024) for PLGA-based systems [43]. Excess drugs may also compromise NP stability, as unencapsulated or surface-associated drug molecules are more susceptible to environmental conditions, such as pH and ionic strength, potentially leading to aggregation or precipitation and reduced bioavailability [44].

One of the major challenges in nanotechnology-based drug delivery systems is the generally low loading capacity of hydrophobic drugs, with reported DL% values frequently below 20% [44,45,46]. In this context, AC8-NPs not only maintained a favorable balance between DL% and EE% but also substantially exceeded the 20% threshold for DL%. This highlights the potential of this formulation to outperform conventional nanocarriers. Moreover, the formulation retained stable particle size and distribution, providing a solid foundation for further in vitro efficacy studies. These results support the selection of AC8-NPs as the optimal formulation for further investigation.

### 3.4. Cytotoxicity

According to Hseu et al. [47], the IC_50_ value of free AC was 136 μg/mL for MDA-MB-231 cells and 316 μg/mL for MCF-7 cells, indicating low anticancer activity in its unmodified form. This limitation could be attributed to its low solubility, which reduces its cellular uptake and diminishes its anticancer efficacy. In this study, loading of AC into chitosan NPs (AC-NPs) reduced the IC_50_ values to 46.9 μg/mL and 75.6 μg/mL for the respective cell lines. This result was consistent with the findings of Chang et al. [48], who reported that MDA-MB-231, a TNBC cell line, exhibited greater sensitivity to specific bioactive compounds isolated from AC. Likewise, Anand et al. [49] reported that nanocurcumin exhibited higher cytotoxicity and bioavailability than free curcumin, suggesting a general mechanism for efficacy enhancement via nanocarrier-mediated delivery.

Notably, AC-NPs were more cytotoxic to the TNBC cell line MDA-MB-231 than to MCF-7 cells, possibly owing to their higher endocytic activity and greater sensitivity to apoptotic signaling. As TNBC lacks effective targeted therapies and tends to develop drug resistance [50,51], the high cytotoxicity of AC-NPs against MDA-MB-231 cells suggests their potential as an adjuvant or alternative therapeutic agent for this type of cancer.

Future studies should elucidate the molecular mechanisms underlying AC-NP-induced apoptosis or cell cycle arrest, such as caspase-3/9 activation, Bax/Bcl-2 expression ratio, and reactive oxygen species production [52,53]. In addition, drug efficacy and toxicological evaluations should be performed in animal models to examine parameters such as blood retention time, tumor regression, organ toxicity, and tissue distribution. Target-modification strategies such as ligand conjugation with CD44 or EGFR can further improve tumor selectivity and therapeutic efficiency.

### 3.5. In Vitro Cellular Uptake Analysis

We investigated the uptake of Fl-AC-NPs by the MDA-MB-231 and MCF-7 breast cancer cell lines using flow cytometry and fluorescence microscopy. The results highlight notable time-dependent uptake patterns and differences between the two cell types. Fl-AC-NPs were rapidly internalized, particularly by MDA-MB-231 cells, with distinct fluorescence observed as early as 15 min post-treatment (Figure 5). This suggests that MDA-MB-231 cells possess a greater NP uptake capacity, potentially due to their greater invasiveness and higher cell membrane fluidity [54,55,56].

Using flow cytometry (Figure 5 and Figure 7), we demonstrated that although the internalization of NPs by MCF-7 cells within 15 min was lower than that by MDA-MB-231 cells, the AC-NP formulation exhibited a substantial inhibitory effect on MCF-7 cells. This phenomenon may be attributed to the multiple favorable characteristics of NPs. On the one hand, the surface of NPs with HA can specifically bind to the overexpressed CD44 receptor on the surface of MCF-7 cells. This enhances the adhesion and accumulation of particles on the cell surface, thereby facilitating endocytosis and cytotoxic effects [57].

In contrast, the AC-NPs formulated in this study had stable, moderate particle sizes ranging from approximately 15 to 80 nm. According to a review report by Deng et al. (2021) [58], several studies have reported that smaller NPs (such as 20–100 nm) not only have a higher tendency to leak through the enhanced permeability and retention effect of tumor blood vessels, but also exhibit a higher intracellular uptake rate, further enhancing their anticancer effect.

Therefore, although early endocytosis of AC-NPs in MCF-7 cells was less than that observed in MDA-MB-231 cells, given the affinity of HA-CD44 and the appropriate particle size design, AC-NPs effectively promoted particle internalization and exerted a substantial inhibitory effect. This result highlights the potential of this NP system for “bidirectional targeting,” given that it not only exploits the high endocytosis characteristic of TNBC cells but also enhances the delivery efficiency to hormone receptor-positive cells through surface receptor affinity.

To further clarify the internalization pathways and their relationship with drug delivery efficiency, targeting the inhibition of endocytosis-related proteins, such as clathrin heavy chain and CAV1, using small interfering RNA (siRNA) may be informative [55,56,59,60]. Additionally, in vivo fluorescence imaging or high-content analysis could facilitate real-time tracking and quantification of the uptake process, providing deeper insights into NP-cell interactions. Future studies assessing glycolytic capacity and reserve may further clarify the link between nanoparticle uptake and cellular metabolism.

## 4. Materials and Methods

### 4.1. Materials

NMuMG murine mammary gland cells, MDA-MB-231 human breast cancer cells, and MCF-7 human breast epithelial cells were obtained from the American Type Culture Collection. Dulbecco’s modified Eagle’s medium (DMEM) was purchased from Sigma-Aldrich (Tokyo, Japan). Ethanol, paraformaldehyde, and Hoechst 33342 were purchased from FUJIFILM Wako Pure Chemicals (Tokyo, Japan). Phosphate-buffered saline (PBS) and SF cell count reagent were purchased from Nissui Pharmaceutical (Tokyo, Japan) and Nacalai Tesque (Kyoto, Japan), respectively.

The AC extract, AC-loaded NPs, and UL-NPs (drug-free) were provided by ROHER Technology Co. (Taichung, Taiwan). NPs comprising chitosan, alginate, and HA were manufactured using a proprietary formulation of the company. A total of 27 g of these three polymers was used as the base material for NP synthesis. Hereafter, this NP system is referred to as a CHA-based carrier. The NPs were named according to the amount of AC added during synthesis, as listed in Table 1.

### 4.2. Characterization of Prepared NPs

SEM and TEM were used to characterize the surface morphology and size of NPs. The NPs were then diluted with deionized water. After drying the NP suspension on the sample stage, the samples were sputter-coated with osmium (MSP-1S; Vacuum Device, Ibaraki, Japan) and observed using SEM (JSM-7800F; JEOL, Tokyo, Japan) at 5 kV. To assess stability, SEM images were acquired at defined storage intervals under identical conditions, and NP morphology and size were compared across time points. For TEM observations, the NP suspension was dropped onto a copper grid (ELS-C10 STEM Cu100P; Okenshoji Co., Ltd., Tokyo, Japan), air-dried, and imaged using TEM (H-7650; Hitachi Ltd., Tokyo, Japan) at an accelerating voltage of 80 kV.

The particle diameters were measured from TEM images using the ImageJ software (ver. 1.54p), with at least 120 particles analyzed per sample. The results are presented as size distribution histograms with mean diameter ± standard deviation (SD).

FTIR spectroscopy was used to confirm the successful encapsulation of AC. FTIR spectra of the AC extract and AC-loaded NPs were measured from 400 to 4000 cm^−1^ using an ATR-FTIR spectrometer (Thermo Fisher Scientific, Madison, WI, USA).

### 4.3. EE% and DL% of Prepared NPs

The NPs were appropriately diluted with deionized water and quantified using a spectrophotometer (UV-2900; Hitachi, Tokyo, Japan) over the range of 250–600 nm. To evaluate the enzymatic degradation of the chitosan NPs, a commercial enzyme blend (Cat. No. 83502; Deerland Probiotics and Enzymes, Kennesaw, GA, USA), which contains cellulase AN as one of its active components, was used. Enzymatic digestion was performed to break down the NPs, adding 0.5 g of enzyme to 3 mL of the AC-NP suspension. The mixture was incubated at 25 °C for 24 h under static conditions. Thereafter, the samples were centrifuged at 8000 rpm for 10 min to separate the residual NPs from the enzyme solution. After removing the enzyme solution, the AC was dissolved in dimethyl sulfoxide (DMSO) and centrifuged again to separate the carriers. The resulting supernatant was diluted with DMSO (ratio of 1:3) and measured at 307 nm using a spectrophotometer, with reference to a calibration curve prepared from pure AC dissolved in DMSO. The calibration curve was linear in the range of 0–1 mg/mL. The *EE*% was calculated as the amount of AC encapsulated in the NPs relative to the initial amount of AC added. The *DL*% was calculated as the amount of encapsulated AC relative to the total mass of NPs. Calculations were performed using Equations (1) and (2):

(1) $$EE\%=\frac{Amount\;of\;AC\;in\;the\;AC\;NPs}{Initial\;mass\;of\;AC}\;\times 100$$

(2) $$DL\%=\frac{Amount\;of\;AC\;in\;the\;AC\;NPs}{Total\;mass\;of\;the\;AC-NPs\;}\times 100$$

### 4.4. Cell Culture

NMuMG cells were cultured in DMEM (Sigma-Aldrich) supplemented with 10% fetal bovine serum (FBS; Atlas Biologicals Inc., Fort Collins, CO, USA). MDA-MB-231 and MCF-7 cells were cultured in DMEM supplemented with 10% FBS, 1% L-glutamine, and 1% penicillin/streptomycin (Nacalai, Kyoto, Japan). All cultures were maintained at 37 °C in a humidified atmosphere containing 5% CO_2_. For all experiments, the cells were counted and seeded at equal initial densities to ensure consistency.

### 4.5. Cell Viability Assay

The cytotoxicity of AC8-NPs was determined using a WST-8 assay (Cell Count Reagent SF Kit; Nacalai, Kyoto, Japan). Cells were seeded in 96-well plates at a density of 1 × 10^4^ cells/well and incubated overnight to allow cell adhesion. Thereafter, the cells were cultured in maintenance medium containing 10% FBS at 37 °C in a 5% CO_2_ atmosphere. Cells were incubated with final concentrations of AC (13.4, 26.8, 53.6, 107.3, and 214.7 μg/mL), delivered either as AC8-NPs or free AC extract, for 24, 48, and 72 h, respectively. After treatment, 10 µL dye solution was added to each well and incubated for 2 h. The absorbance was measured at 450 nm using a microplate reader (Multiskan GO; Thermo Fisher Scientific, Waltham, MA, USA).

### 4.6. Preparation of Fluorescent AC-NP Conjugates

To conjugate fluorescein to amine groups on the NP surface, 1 mL of AC8-NP suspension in PBS (pH 7.4) was mixed with 10 µL of NHS fluorescein solution (0.1 M in DMSO; Thermo Fisher Scientific K.K., Tokyo, Japan). The mixture was then shaken at room temperature for 2 h in the dark. Unreacted NHS fluorescein was removed by centrifugation, and the cells were washed twice with PBS. The resulting conjugates (Fl-AC-NPs) were examined using a confocal microscope (Olympus IX-81; Olympus Optical Co., Ltd., Tokyo, Japan) to confirm successful conjugation of fluorescein to the NPs.

### 4.7. Visualization of AC-NP Cellular Uptake Using Confocal Microscopy

Cells were inoculated at a density of 1 × 10^5^ cells in a 35 mm glass-bottom dish with 2 mL of medium and cultured overnight at 37 °C. Subsequently, 50 μL of Fl-AC-NPs (at an original concentration of 11.0 mg/mL, corresponding to an AC concentration of approximately 3.50 mg/mL) was added, and the cells were incubated for 0.5, 1, 6, or 24 h. Subsequently, the cells were washed twice with PBS to remove residual NPs and fixed with 4% paraformaldehyde in PBS. Following fixation and washing twice with PBS, the nuclei were stained with Hoechst 33342 (1:2000 dilution in PBS; Dojindo, Kumamoto, Japan) for 15 min at room temperature in the dark. The intracellular distribution and uptake of the NPs were visualized using confocal microscopy. Untreated cells were used as controls.

### 4.8. Quantification of AC-NP Internalization by Flow Cytometry

Flow cytometry was performed to quantify the cellular uptake of Fl-AC-NPs. Cells (2 × 10^5^ cells/well) were seeded in 6-well plates and cultured overnight at 37 °C. Cells were then treated with 50 μL of Fl-AC-NPs (at an original concentration of 11.0 mg/mL, corresponding to an AC concentration of approximately 3.50 mg/mL) and incubated for 0.25, 1, 2, 6, or 24 h. Untreated cells served as controls. After incubation, the cells were washed twice with PBS, trypsinized, and resuspended in PBS. Fluorescence was measured using a FACSymphony A1 (Becton Dickinson, San Jose, CA, USA). Forward and side scatter were used to gate live cells and exclude debris, respectively. A total of 5000 fluorescence-positive cells per sample were analyzed.

### 4.9. Statistical Analyses

Data are presented as the mean ± SD (*n* = 3). Tukey’s test was used to compare differences between groups, with statistical significance indicated as * *p* < 0.05 and ** *p* < 0.001.

## 5. Conclusions

CHA-based AC-NPs were successfully developed to enhance the solubility, stability, and intracellular uptake of the active components derived from AC in an in vitro breast cancer cell model. Compared with the free form, AC-NPs substantially reduced the IC_50_ values in both MDA-MB-231 and MCF-7 cells, demonstrating enhanced cytotoxicity. Notably, the TNBC and highly invasive MDA-MB-231 cell lines exhibited greater sensitivity to AC-NPs, highlighting their potential for precision cancer therapy. Flow cytometry and fluorescence microscopy revealed rapid cellular uptake of fluorescein-labeled NPs and time-dependent accumulation within cells, with MDA-MB-231 cells exhibiting the highest uptake capacity. This may be associated with variations in surface receptor profiles, endocytic pathways, and intracellular metabolic rates, consistent with previous reports that TNBC cells exhibit elevated endocytosis and exocytosis. In summary, AC-NPs effectively addressed the limitations of natural compound potency while exhibiting favorable DL% efficiency, cellular uptake, and anticancer activity. Future investigations should explore the specific endocytic pathways involved, such as clathrin- or caveolin-mediated endocytosis, potentially employing siRNA to target key endocytic proteins, and validate the internalization mechanism. Moreover, in vivo fluorescence imaging and high-content analysis should be conducted to further elucidate the dynamic behavior of NPs within cells.

## Figures and Tables

**Table 1:** Composition and description of AC-loaded nanoparticle formulations.

Name	AC Input Amount (g)	Description
Unloaded-NPs (UL-NPs)	0	Nanoparticles without AC (blank control)
AC8-NPs	8	Nanoparticles loaded with 8 g of AC extract
AC12-NPs	12	Nanoparticles loaded with 12 g of AC extract
AC16-NPs	16	Nanoparticles loaded with 16 g of AC extract

AC, *Antrodia camphorate*; Fl-AC-NPs, fluorescein-labeled AC-NPs; NPs, nanoparticles.

## Data Availability

The original contributions presented in this study are included in the article/Supplementary Materials. Further inquiries can be directed to the corresponding author.

## Supplementary Materials

The following supporting information can be downloaded at: https://www.mdpi.com/article/10.3390/ijms26178420/s1.

## Abbreviations

The following abbreviations are used in this manuscript:

AC	*Antrodia camphorata*
DL	Drug loading
DMEM	Dulbecco’s modified Eagle’s medium
EE	Encapsulation efficiency
ER	Estrogen receptor
FBS	Fetal bovine serum
FTIR	Fourier-transform infrared
HA	Hyaluronic acid
NPs	Nanoparticles
PBS	Phosphate-buffered saline
SD	Standard deviation
SEM	Scanning electron microscopy
TEM	Transmission electron microscopy
TNBC	Triple-negative breast cancer
