# Supplementary material for: Natural Polysaccharide-Based Nanoparticles Enhance Intracellular Delivery and Cytotoxicity of Antrodia camphorata in Breast Cancer Cells
Source: Int J Mol Sci. 2025 Aug 29;26(17):8420. doi: 10.3390/ijms26178420 (PMC12428654; doi:10.3390/ijms26178420)
Supplement: Supplementary file 1 [file ijms-26-08420-s001.zip › ijms-3804845-supplementary.pdf]

Supporting Materials

Natural Polysaccharide-Based Nanoparticles Enhance the Intracellular Delivery and Cytotoxicity of Antrodia camphorata in Breast Cancer Cells

Yu-Chen Tsai[1], Hiroki Miyajima[1], Ming-Yang Chou[2*], and Satoshi Fujita[1*]

[1]Department of Frontier Fiber Technology and Sciences, University of Fukui, Fukui, 910-8507, Japan

[2] ROHER Technology Co., Taichung, 41141, Taiwan, Republic of China.

*Correspondence: rohertech007@gmail.com (M.Y.C.); fujitas@u-fukui.ac.jp (S.F); Tel.: +81-0776-27-9969

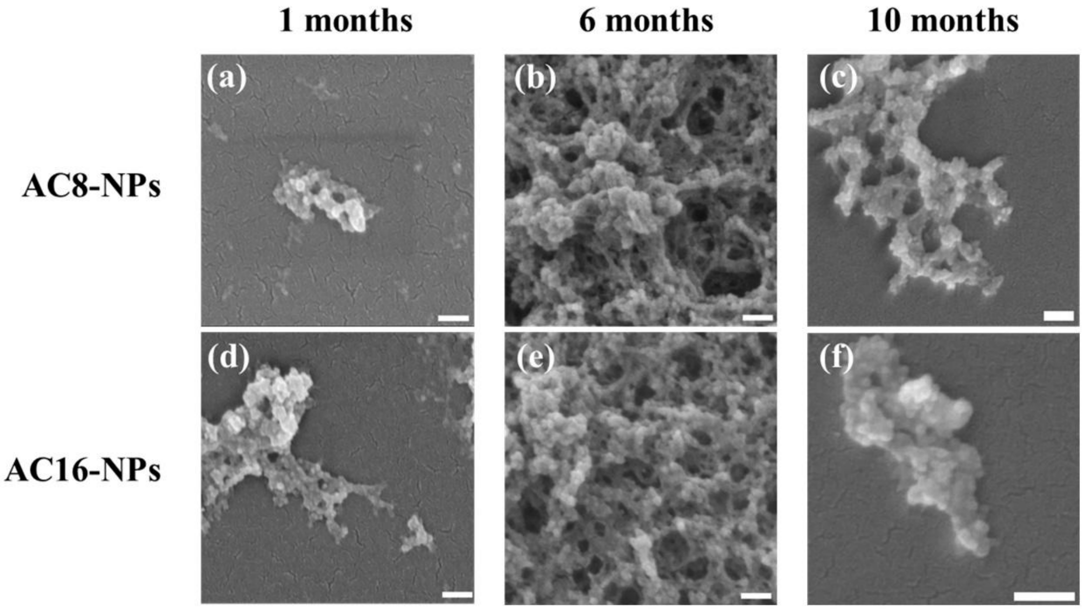

Fig. S1 SEM images of AC-NPs after different storage durations at 25 °C in the dark. Top row: AC8-NPs after (a) 1 month, (b) 6 months, and (c) 10 months. Bottom row: AC16-NPs after (d) 1 month, (e) 6 months, and (f) 10 months. All scale bars = 100 nm.

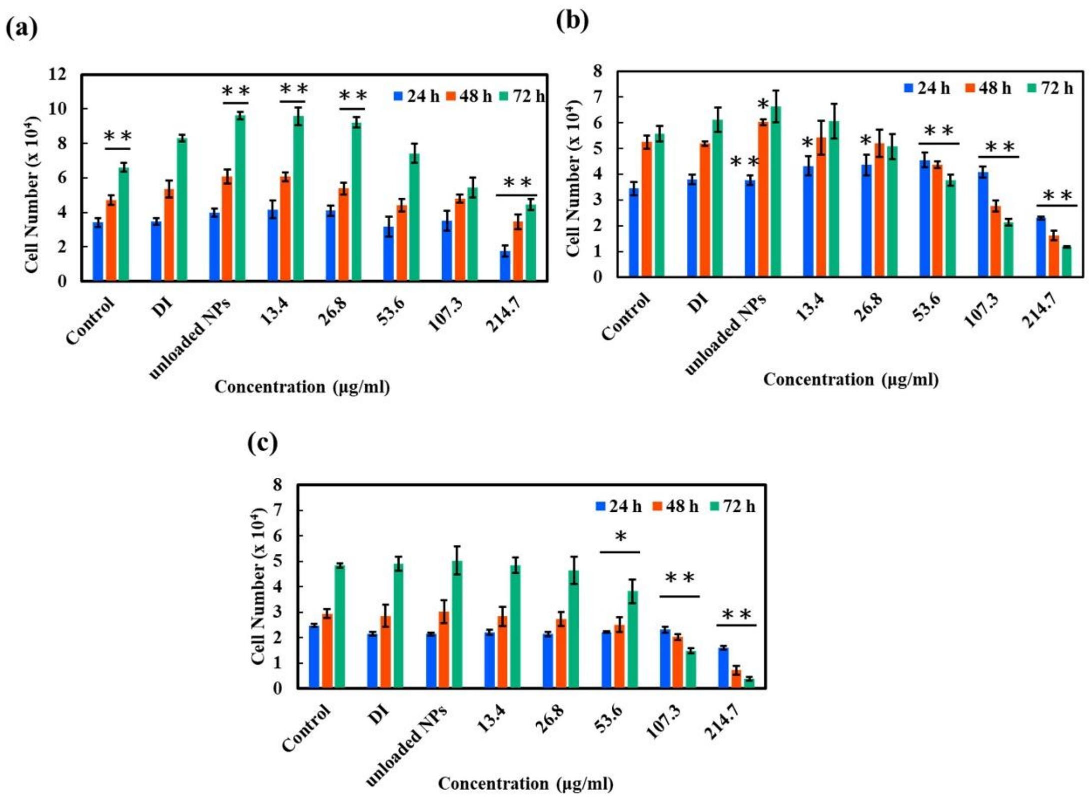

Fig. S2 Cell viability of breast cancer cells treated with UL-NPs and AC-NPs. (a) NMuMG, (b) MDA-MB-231, and (c) MCF-7 cells were incubated with NPs for 24, 48, and 72 h, followed by CCK-8 assay. Absorbance at 450 nm was measured using a spectrophotometer. Data are presented as mean ± SD (*n* = 3).
